# Feigning Amnesia Moderately Impairs Memory for a Mock Crime Video

**DOI:** 10.3389/fpsyg.2018.00625

**Published:** 2018-04-30

**Authors:** Ivan Mangiulli, Kim van Oorsouw, Antonietta Curci, Harald Merckelbach, Marko Jelicic

**Affiliations:** ^1^Department of Education, Psychology, Communication Science, University of Bari Aldo Moro, Bari, Italy; ^2^Forensic Psychology Section, Department of Clinical Psychology, Faculty of Psychology and Neuroscience, Maastricht University, Maastricht, Netherlands

**Keywords:** feigning amnesia, simulation, lack of rehearsal, mock crime video, inner speech

## Abstract

Previous studies showed that feigning amnesia for a crime impairs actual memory for the target event. Lack of rehearsal has been proposed as an explanation for this memory-undermining effect of feigning. The aim of the present study was to replicate and extend previous research adopting a mock crime video instead of a narrative story. We showed participants a video of a violent crime. Next, they were requested to imagine that they had committed this offense and to either feign amnesia or confess the crime. A third condition was included: Participants in the delayed test-only control condition did not receive any instruction. On subsequent recall tests, participants in all three conditions were instructed to report as much information as possible about the offense. On the free recall test, feigning amnesia impaired memory for the video clip, but participants who were asked to feign crime-related amnesia outperformed controls. However, no differences between simulators and confessors were found on both correct cued recollection or on distortion and commission rates. We also explored whether inner speech might modulate memory for the crime. Inner speech traits were not found to be related to the simulating amnesia effect. Theoretical and practical implications of our results are discussed.

## Introduction

Offenders in homicide and sex offense cases often claim crime-related amnesia (Cima et al., 2002, 2004; Pyszora et al., 2003, 2014; Bourget and Whitehurst, 2007). For instance, even though it is hard to determine to which degree defendants may intentionally feign amnesia following a crime, Pyszora et al. (2003) found that 29% of a 1-year cohort of individuals sentenced to life imprisonment claimed memory loss for their deeds (31.4% of those convicted of homicide). While it might be that the intense emotional arousal that some perpetrators experience during the crime might impair memory (e.g., Kopelman, 1995), there is also the distinct possibility that perpetrators feign memory loss (Centor, 1982; Marshall et al., 2005). Although majority of jurisdictions are reluctant to equate amnesia with incompetency, claiming crime-related amnesia in court raises the question whether the defendant’s ability to understand the trial proceedings or his capacity to consult with his attorney are impaired (e.g., Cima et al., 2002; Tysse, 2005; Tysse and Hafemeister, 2006). For that reason, some individuals who are charged with serious crimes pretend to have memory loss for their offense (Christianson and Merckelbach, 2004; Smith and Resnick, 2007; van Oorsouw and Merckelbach, 2010).

Furthermore, when offenders adopt that strategy relevant information might be forgotten as it has been demonstrated that feigning amnesia has a detrimental effect on the genuine memory reported by feigners for those target events (e.g., Christianson and Bylin, 1999; van Oorsouw and Merckelbach, 2004, 2006). Although what perpetrators truly remember about the crime may differ from what they actually select to report or claim to remember, because of the risk of undisclosed information in high-stake cases it is crucial for the legal context to ascertain how people remember remarkable information over time despite having previously feigned amnesia (Porter et al., 2001; Bourget and Whitehurst, 2007).

Several studies have shown that feigning amnesia can undermine actual memory for a crime (Christianson and Bylin, 1999; Bylin, 2002; Bylin and Christianson, 2002; van Oorsouw and Merckelbach, 2004, 2006; Sun et al., 2009; Mangiulli et al., unpublished). The typical procedure to investigate the memory-undermining effect of simulating crime-related amnesia is as follows. First, participants are usually exposed to a written/narrative story about a crime and instructed to identify themselves with the perpetrator or asked to commit a mock crime. Next, participants are assigned to one of the two conditions: Some participants are asked to comply with the police by reporting as accurately as possible all information they remember about the event (i.e., further referred to as confessors); some others are instructed to minimize or evade their responsibility for the crime by feigning memory loss for the offense (i.e., referred to as simulators; Christianson and Bylin, 1999; Bylin, 2002; van Oorsouw and Merckelbach, 2006). Sometimes, a third condition is included (Bylin and Christianson, 2002; van Oorsouw and Merckelbach, 2004; Sun et al., 2009; Mangiulli et al., unpublished) consisting of participants who do not receive any instruction, and who serve as a delayed test-only control condition (i.e., further referred to as controls). In these studies, during the first memory phase, participants in the first two conditions (confessors vs. simulators) were given a free and cued recall test pertaining to the crime. One week later, during the second memory phase, all participants of the two (or three) conditions were requested to genuinely report about the crime event, through the same free and cued recall test. Typically, participants initially instructed to feign amnesia show a poorer memory for their crime than those who were asked to confess it (Christianson and Bylin, 1999; Bylin, 2002; Bylin and Christianson, 2002; van Oorsouw and Merckelbach, 2004; Sun et al., 2009; Mangiulli et al., unpublished). Thus, research on feigning amnesia suggests that simulating memory loss impairs actual memory for a crime (e.g., Christianson and Bylin, 1999; Bylin, 2002; van Oorsouw and Merckelbach, 2004). Furthermore, when a delayed test-only control condition was included in the experimental design, no significant differences in memory performance were observed between controls and simulators 1 week after either being exposed to or committing the crime (e.g., Bylin and Christianson, 2002; van Oorsouw and Merckelbach, 2004). In contrast to confessors, simulators and controls did not have to provide details of the crime just after the crime stimulus, namely they did not engage in rehearsing the crime. This lack of rehearsal, therefore, might explain the memory-undermining effect of feigning amnesia (see van Oorsouw and Merckelbach, 2004).

Relatedly, it is commonly observed that feigning participants comply with their instructions by withholding, distorting, and introducing new information (i.e., commission errors) on the initial memory test (Bylin, 2002; Bylin and Christianson, 2002; van Oorsouw and Merckelbach, 2004, 2006). Clearly, simulating participants use laypeople’s ideas about how feign amnesia works (Bylin, 2002). Thus, even though feigning amnesia might mostly lead to omissions (e.g., Christianson and Bylin, 1999; van Oorsouw and Merckelbach, 2004), it is not surprising that van Oorsouw and Giesbrecht (2008) found that participants initially instructed to minimize culpability for a mock crime increased commission errors over time, as compared with genuinely responding controls. Accordingly, the act of coming up with a personal, self-generated story of the crime (i.e., simulated version of the crime) could enhance errors, but may not affect the number of correct details provided (Chrobak and Zaragoza, 2008, 2012; van Oorsouw and Giesbrecht, 2008; Ackil and Zaragoza, 2011).

More recently, Mangiulli et al. (unpublished) explored whether confronting simulators with visual and verbal cues about a crime – by trying to induce rehearsal of details of the offenses – would prevent impairments in simulators’ memory. With this set-up, simulators performed on a similar level as confessors (when they were prompted with verbal cues), and interestingly they outperformed controls on a memory test for the crime regardless of a rehearsal induction. This indicates that lack of rehearsal indeed does not fully account for feigners’ memory detriments. Unlike previous research in this field (e.g., Bylin and Christianson, 2002; Sun et al., 2009), Mangiulli et al. (unpublished) used a video clip instead of a narrative story as a mock crime. They suggested that compared to a narrative story, a mock crime video was better encoded by participants engaged in role playing, such as feigners and confessors, leading to a better memory performance over time. It is well known, indeed, that visual stimuli are generally remembered better than verbal stimuli (i.e., words, narrative stories) since images are encoded into both verbal and image codes, while words are primarily coded verbally (Paivio, 1976, 1986). Moreover, images are more distinctive in their features and better evoked than words (Nelson et al., 1976; Mintzer and Snodgrass, 1999). Thus, even though controls were exposed to the same crime material, it seems that both confessor and simulator groups actively elaborated upon the crime video so as to provide specific statements concerning their instructions, contributing to a more solid memory trace of the crime event compared with controls. The Mangiulli et al. (unpublished), suggests that the memory-undermining potential of feigning amnesia is more modest and fragile than it has previously been assumed. The results of that study seem to indicate that when using a mock crime video as crime material, the phenomenon is limited to the comparison between confessors and simulators.

Following this line, the main purpose of the present study was to further investigate lack of rehearsal as the best explanation for the memory-undermining effect of simulating amnesia. We replicated the standard procedure to study feigning amnesia effects (e.g., van Oorsouw and Merckelbach, 2004) by using the same mock crime video employed by Mangiulli et al. (unpublished) instead of a narrative mock crime story (e.g., Bylin and Christianson, 2002; Sun et al., 2009). We showed participants a video clip pertaining to a violent crime and asked them to either feign amnesia (simulators group) or confess the crime (confessors group) during the first memory phase. We also included a delayed test-only control group consisting of participants who did not receive any instruction. After 1 week, we requested all three groups to genuinely report all the information they could remember about the offense. We expected that simulators would recollect fewer correct details of the crime than confessors (hypothesis 1). However, we anticipated that both confessors and simulators would perform better than the delayed-test only control group (hypothesis 2). Finally, we predicted simulators to report more distortion and commission errors (i.e., introduction of new information) than confessors on the subsequent memory recall (hypothesis 3 and 4, respectively).

Moreover, we attempted to extend the study by Mangiulli et al. (unpublished) by investigating whether the memory-undermining effect of feigning amnesia is modulated by inner speech activity. Inner speech refers to the subvocal rehearsing of personal events (Alderson-Day and Fernyhough, 2015) and includes various characteristics such as dialogicality and condensation, the presence of other people voice and evaluative/motivational inner speech (McCarthy-Jones and Fernyhough, 2011; Alderson-Day et al., 2014; Alderson-Day and Fernyhough, 2015). For instance, the use of evaluative/motivational inner speech such as “*I should do this*,” might be linked to the feigners’ inclination in being consistent with their own simulated version of the crime in distinct circumstances (e.g., during preliminary investigations). Yet, perpetrators might estimate their deeds by engaging themselves in a self-evaluative-talk. Accordingly, common contents of inner speech refer to self- addressed evaluations and emotional states, in which continued inner speaking would regularly refresh experiences and maintain the corresponding memory traces in an “inner loop” (Alderson-Day and Fernyhough, 2015). Thus, simulators might internally think of the offense they perpetrated like entailing consequences for the event. By doing so, they might feed the actual memory of the crime. If this was the case, we would expect a significant correlation between the individual inner speech traits and the memory undermining effect of feigning.

## Materials and Methods

### Participants and Design

The present study was approved by the standing Ethical Committee of the Faculty of Psychology and Neuroscience, Maastricht University (ERCPN application - 167 06 05 2016). Using a snowballing sampling technique (Goodman, 1961), we tested 111 individuals who volunteered to take part in the study (range 18–58, *M*_age_ = 22.60, *SD* = 9.64; 70% women). Participants were randomly assigned to one of the three conditions – simulators (*N* = 37), confessors (*N* = 37), and controls (*N* = 37). The study used a 3 × 2 mixed model design with condition (simulators vs. confessors vs. controls) as between subjects variable, and memory test–retest (T1 vs. T2) as a within subjects repeated measure variable. The dependent variable was the proportion of correctly recollected information in a free and cued recall test. Furthermore, we calculated distortion and commission errors generated during memory tests.

### Materials and Procedure

#### Pre-experimental Phase

Participants were tested in a quiet room. The study consisted of two phases. During the pre-experimental phase, each participant was invited to complete the Structured Inventory of Malingered Symptomatology (SIMS; Smith and Burger, 1997), and the Varieties of Inner Speech Questionnaire (VISQ; McCarthy-Jones and Fernyhough, 2011). This last instrument was administered to explore the relation between inner speech and the memory undermining effect of simulating. In order to guarantee homogeneity in our sample before the experimental phase, the SIMS was assessed to check for possible differences among groups with regard to their feigning tendency.

#### Structured Inventory of Malingered Symptomatology (SIMS; Smith and Burger, 1997)

The SIMS^1^ is a two option self-report measure to screen for over-reporting of mental symptoms and consists of a 75 items which are divided into five subscales (affective disorders; amnestic disorders; low intelligence; neurological impairment; psychosis). It includes items asking for atypical symptoms (e.g., *“Walking is difficult for me because of my problems with balance”*). Answers indicative of over-reporting are summed to obtain a total SIMS score (α = 0.73).

#### Varieties of Inner Speech Questionnaire (VISQ; McCarthy-Jones and Fernyhough, 2011)

The VISQ is an 18 item self-report instrument measuring the phenomenological proprieties of inner speech along four dimensions: Condensed (α = 0.72; *“I think to myself in words using brief phrases and single words rather than full sentences”*); Dialogic (α = 0.85; *“I talk back and forward to myself in my mind about things”*); Other People (α = 0.85; *“I experience the voices of other people asking questions in my head”*); Evaluative/Motivational (α = 0.76; *“I experience the voices of other people asking questions in my head”*). Participants have to rate a 6-point Likert scale anchoring from “certainly does not apply to me” (1) to “certainly applies to me” (6).

#### Mock Crime Video

After the pre-experimental phase, all participants were requested to pay attention to the mock crime video and were instructed to identify themselves with the character that appeared on the scene first (i.e., offender). The crime contained a violent scene between two armed men (2.30 min): A man entering a restroom was attacked by another man. After a severe fight, the attacker strangled the victim with his belt leaving him lifeless on the ground. After the exposure to the mock crime video, all participants were given a 10-min distractor task (i.e., computer game). This task was administered to avoid the possible ceiling effect in the following memory tests (Bylin, 2002).

#### Memory Test Phase (T1)

Next, participants belonging to the simulator and confessor groups received the following instruction: *“Imagine being the offender. Imagine that you have been arrested because you are the prime suspect of the murder. That day, a witness saw you there and all the evidence points to you*. *Right now, a policeman is asking you to tell what happened.”* Following previous studies (e.g., Bylin and Christianson, 2002; van Oorsouw and Merckelbach, 2004; Sun et al., 2009; Mangiulli et al., unpublished) free and cued recall tests were employed as memory measures. Through a free recall, participants were invited to report their statements in accordance with the condition to which they were assigned. That is, simulators were instructed to report the crime as if they could not properly remember what happened. To evade punishment, simulating participants were free to omit, distort or report other information. Confessors, on the other hand, were asked to honestly report details about the crime in order to collaborate with the police. After this free recall, participants in both groups were given 14 cued recall questions concerning the mock crime video and were instructed to answer them by adhering to the instruction previously given (i.e., simulating or confessing). In line with previous research (e.g., Bylin and Christianson, 2002; van Oorsouw and Merckelbach, 2004; Sun et al., 2009), and in contrast to simulators and confessors, controls were not given a memory test. Although they were asked to identify themselves with the offender, participants in the control condition did not receive any instruction after the mock crime viewing and they were directly scheduled for the second session.

#### Memory retest phase (T2)

After 1 week, all participants – including controls – were specifically requested to be as specific as possible while providing all the information regarding the mock crime, as if they had decided to collaborate with the police. Contrary to the instruction received during the first memory phase, this time simulators were instructed to give up their role as feigner and recollect all they could remember about the target event. Confessors again received the instruction to comply with the police by reporting each and every detail about the mock crime video. Similarly, controls were now asked to recollect as much as they could remember about the criminal act. In the cued recall task, all participants were told to honestly answer the 14 questions. Finally, participants were thanked and debriefed.

### Memory Test–Retest Scoring

#### Free Recall

Following Mangiulli et al. (unpublished), a scoring system was established to assess participants’ free recall. We first classified the mock crime video into 50 critical information units. Critical information was defined as a relevant piece of the video. For each correct unit of information reported (e.g., *“I assaulted the victim from the back”*), participants scored 1 point (maximum = 50). Moreover, participants earned a half point for each partially correct unit of information given (e.g., *“I assaulted the victim”*). In line with previous studies (i.e., van Oorsouw and Merckelbach, 2004; Sun et al., 2009), the entire correct score was transformed into proportions (range = 0–1) by dividing the number correct units reported by the maximum obtainable score. Additionally, we identified the number of distorted units (e.g., *“I killed the victim by shooting him”*) and commissions (i.e., introduction of new information that was not displayed in the video: *“The victim was wearing a ski mask”*). The first author and two assistants, who were blind to the study conditions, scored participants’ free recall. The interclass correlation coefficient (ICC) average measure for the number of correct free recall scores was 0.93 (*p* < 0.001); the ICC’s for distortions and commissions were 0.86 and 0.83, respectively (both *p*_s_ < 0.001).

#### Cued Recall

The cued recall test consisted of fourteen questions regarding both central (seven questions; i.e., weapon or blood), and peripheral details (seven questions; i.e., characters’ clothing, details about the location) of the mock crime video. Participants earned 1 point for each correct answer given (e.g., Question: *“Where did the murder take place?,” “In the parking lot’s toilet”*). Again, a half point was awarded for a partial correct answer (e.g., *“In the toilet”*). No penalty was given when participants did not provide any answer (e.g., *“I do not remember”*). The maximum obtainable score was 14. Similar to the free recall, the total cued recall score was transformed into proportions (range = 0–1). Furthermore, the number of distorted details and commissions were identified (e.g., *“The murder took place near a fire extinguisher in the parking lot,”* and *“The murder took place in a bar,”* respectively). The ICC average measure for the number of correct cued recall information was 0.96 (*p* < 0.001); the ICC average measure of distortions and commissions for cued recall was 0.88 and 0.79, respectively (both *p*_s_ < 0.001).

## Results

### Manipulation Check on Feigning Tendency

A one way ANOVA was conducted on SIMS total score to exclude possible individual differences in the feigning tendency among three groups^2^ before the experimental phase. The main effect of condition was found not significant, *F*(2,106) = 0.03, *p* = 0.971, so that participants did not differ as to their simulating predisposition.

### Free Recall – Correctness Scores

A 2 × 2 repeated measures ANOVA with condition (simulators vs. confessors) as a between subjects factor and memory test–retest (T1 vs. T2) as a within subjects factor was run on the free recall correctness score. The main effects of condition and memory test–retest were found significant, *F*(1,71) = 60.62, *p* < 0.001, $\eta_{p}^{2}$ = 0.46, and *F*(1,71) = 8.86, *p* = 0.004, $\eta_{p}^{2}$ = 0.11, respectively. The significant condition by test–retest interaction, *F*(1,71) = 24.66, *p* < 0.001, $\eta_{p}^{2}$ = 0.26, showed that simulating participants reported more correct information at T2 than T1, *t*(35) = 7.83, *p* < 0.001, *d* = 2.00. This indicates that participants in the simulation condition properly followed their instruction. No difference was found for confessors in the proportion of correct information reported at the two memory phases, *t*(36) = 1.16, *p* = 0.25.

In order to observe differences in the correctness score among all conditions of the design (simulators vs. confessors vs. controls), a one-way ANOVA was conducted only on the retest memory phase (T2). The main effect of condition reached significance, *F*(2,107) = 17.74, *p* < 0.001, $\eta_{p}^{2}$ = 0.25. *Post hoc* test with Bonferroni correction indicated that simulators reported less correct information than confessors, *p* = 0.014, 95% CI [-4.22 -0.36], *d* = 0.61. As expected, and in line with our hypothesis (Hp. 1), feigning amnesia undermined memory for the mock crime video. Furthermore, in line with our prediction (Hp. 2), both simulators and confessors were able to recall significantly more correct information than controls, *p* = 0.009, 95% CI [0.47 0.43], *d* = 0.83, and *p* < 0.001, 95% CI [2.77 6.60] *d* = 1.36, respectively. Proportions of free recall correctness^3^ are shown in **Table 1**.

**Table 1:** Free and cued recall proportions for each condition during the first (T1) and the second (T2) memory phase.

Memory phase	Simulators		Confessors		Controls
	T1	T2	T1	T2	T2
**Free recall**					
Correct	0.04^a^ (0.03)	0.13^a,b,c^ (0.06)	0.19 (0.09)	0.17^b,d^ (0.08)	0.07^c,d^ (0.04)
Distortion^∗^	0.81^e^ (0.92)	2.03^e^ (1.30)	1.81 (1.24)	1.62 (1.67)	1.78 (1.31)
Commission^∗^	0.89^f^ (1.19)	0.14^f^ (0.42)	0.11 (0.39)	0.03 (0.16)	0.03 (0.16)
**Cued recall**					
Correct	0.10^a^ (0.05)	0.16^a^ (0.04)	0.18 (0.04)	0.18^b^ (0.04)	0.15^b^ (0.03)
Distortion^∗^	2.77 (1.73)	2.76 (1.62)	2.86 (1.62)	2.90 (1.80)	3.90 (1.63)
Commission^∗^	0.35^c^ (0.67)	0.05^c,d^ (0.23)	0.22^e^ (0.58)	0.10^e,f^ (0.31)	0.22^d,e,f^ (0.38)

*Standard deviations are shown between parentheses. ^∗^Distortion and commission errors are displayed in absolute numbers. Same letters within the same row display significant differences between groups at *p* < 0.05.*

### Free Recall – Distortion and Commission Errors

Two 2 × 2 repeated measures ANOVAs with condition (simulators vs. confessors) as a between subjects factor and test–retest (T1 vs. T2) as a within subjects factor were separately performed on distortion and commission errors. Regarding the distortions rate, the main effect test–retest, and the interaction effect condition by test–retest reached significance, *F*(1,71) = 6.46, *p* = 0.013, $\eta_{p}^{2}$ = 0.08, and *F*(1,71) = 12.06, *p* = 0.001, $\eta_{p}^{2}$ = 0.14, respectively. By contrast, the main effect of condition was not found to be significant, *F*(1,71) = 1.68, *p* = 0.20. Surprisingly, simulators provided more distorted details at T2 than T1, while confessors did not over time, *t*(35) = 5.50, *p* < 0.001, *d* = 1.10, and *t*(36) = -0.561, *p* = 0.58, respectively. Unexpectedly, and contrary to our hypothesis (Hp. 3), no significant differences were found between simulators and confessors at T2 on the number of distorted details provided, *t*(71) = 1.16, *p* = 0.25, *d* = 0.27.

With regard to the commission errors, the main effects of condition and memory test–retest were found to be significant, *F*(1,71) = 16.30, *p* < 0.001, $\eta_{p}^{2}$ = 0.19, and *F*(1,71) = 14.55, *p* < 0.001, $\eta_{p}^{2}$ = 0.17, respectively. Moreover, the condition by memory test–retest interaction effect was analyzed, *F*(1,71) = 9.42, *p* = 0.003, $\eta_{p}^{2}$ = 0.12. Simulators significantly provided fewer commissions at T2 compared to T1, while no differences were found in confessors between the two memory phases, *t*(35) = 3.60, *p* = 0.001, *d* = 0.81, and *t*(36) = -1.14, *p* = 0.26, respectively. Against our assumption (Hp. 4), simulators did not significantly differ from confessors at T2 on the number of commissions reported, *t*(71) = 1.49, *p* = 0.14, *d* = 0.34.

Finally, two one-way ANOVAs were independently run on distortion and commission errors to investigate differences among groups (simulators vs. confessors vs. control) during T2. The main effect of condition did not reach significance with respect to both distortions and commissions, *F*(2,107) = 0.73, *p* = 0.482, and *F*(2,107) = 1.96, *p* = 0.145, respectively. Absolute numbers for both distorted details and commission errors are displayed in **Table 1**.

### Cued Recall – Correctness Scores

In line with the free recall analyses, an identical pattern of ANOVAs was conducted on the cued recall correctness scores. The interaction effect condition by memory test–retest, *F*(1,72) = 27.91, *p* < 0.001, $\eta_{p}^{2}$ = 0.28, showed that simulators reported more correct information units at T2 than at T1, which was in line with their instruction, *t*(36) = 6.23, *p* < 0.001, *d* = 1.09. However, confessors did not differ in the amount of correct information reported from T1 to T2, *t*(36) = -0.31, *p* = 0.76. Interestingly, and in contrast to our hypothesis (Hp. 1), a *post hoc* test with Bonferroni correction showed no significant difference between simulators and confessors with respect to the number of correct details during T2, *p* = 0.128, 95% CI [-1.98.16], *d* = 0.44. Thus, feigning memory loss did not impair genuine memory for a mock crime when participants were requested to honestly recollect the target event through a cued recall test.

Moreover, partially supporting our prediction (Hp. 2), only confessors reported more correct details than controls since no significant differences were found between simulators and controls at T2, *p* = 0.03, 95% CI [0.07 2.22], *d* = 0.64, and *p* = 1.00, 95% CI [-0.83 1.31], *d* = 0.13, respectively. See **Table 1** for cued recall correctness^4^ proportions.

### Cued Recall – Distortion and Commission Errors

By running the same set of ANOVAs, a pattern comparable to the free recall was observed on the cued recall distortion and commission errors. In contrast with our hypothesis (Hp. 3), no significant differences were found between simulators and confessors with respect to distortions at T2 *t*(72) = 0.34, *p* = 0.73, *d* = 0.08.

Yet, the main effect of time was found significant for the commission errors, *F*(1,72) = 6.20, *p* = 0.015, $\eta_{p}^{2}$ = 0.08, showing that all participants (i.e., simulators and confessors) reduced the number of commission errors from T1 to T2, *t*(73) = 2.48, *p* = 0.01, *d* = 0.53. No other main or interaction effects were found to be significant on commissions, *F*_s_(1,72) < 1.35, *p* > 0.25, $\eta_{p}^{2}$ < 0.02, meaning that simulators did not differ from confessors on commission errors at T2 against our expectation (Hp. 4).

With respect to differences among groups at T2, the main effect of condition was significant for distortion rates, *F*(2,108) = 5.02, *p* = 0.008, $\eta_{p}^{2}$ = 0.08. Bonferroni corrected *post hoc* test revealed that controls reported more distorted details than both simulators and confessors at T2, *p* = 0.01, 95% CI [0.18 2.08], *d* = 0.70, and *p* = 0.03, 95% CI [0.05 1.95], *d* = 0.58, respectively. Finally, no significant main or interaction effects were found on commission errors among groups at T2, *F_s_*(2,108) < 1.73, *p* > 0.18. See **Table 1** for cued recall distorted information and commission errors.

### Simulating Amnesia and Inner Speech

In line with van Oorsouw and Merckelbach (2004), we first computed the simulating amnesia effect for the feigning participants’ free and cued recall performance by calculating difference scores for the memory variables (▲ Free Recall = Free Recall T2 – Free Recall T1; ▲ Cued Recall = Cued Recall T2 – Cued Recall T1). Next, we correlated the simulating amnesia effect with VISQ (*M_Condensed_* = 14.11, *SD* = 5.24; *M_Dialogic_* = 11.38, *SD* = 5.25; *M_OtherPeople_* = 8.31, *SD* = 4.46; *M_Evaluative/Motivational_* = 13.02; *SD* = 4.71). No significant correlations were found between the memory-undermining effects of feigning amnesia and these individual difference traits with respect to both free and cued recall performances, *r*_s_ < 0.12, *p* > 0.47, and *r*_s_ < -0.05, *p* > 0.11, respectively.

## Discussion

The present study aimed to replicate and extend previous research on the feigning amnesia for a mock crime paradigm (e.g., van Oorsouw and Merckelbach, 2004; Mangiulli et al., unpublished) to further study decrements in rehearsal as an explanation for the memory-undermining effect of simulating amnesia. With respect to our first hypothesis, feigning amnesia undermined the actual memory for the criminal event since simulators provided less correct information than confessors on the free recall. However, the memory detrimental effect following feigning of amnesia took place only during the free memory test, since previous simulators and confessors did not differ on the final cued recall test. The same pattern of results was observed on the accuracy score. Hence, feigning amnesia for a mock crime in this study did not lead to the strong memory-undermining effect as shown in previous research (e.g., Christianson and Bylin, 1999; van Oorsouw and Merckelbach, 2004, 2006). Moreover, it seems that the lack of the simulating amnesia effect is related to the memory test that was used, as evidenced by better performance on the cued recall test than on the free recollection test.

Next, according to our second hypothesis, simulating participants did report more correct crime-related information than those who were not interviewed in the first place (i.e., controls) on the free recall test – although no significant differences were found on the accuracy rates between both groups, in which distortion and commissions errors were taken into account. Note that this prediction is particularly important since, based on the absence of significant differences between feigners and controls on the final recall tests, “lack of rehearsal” has been pointed out as the best explanation for the memory-undermining effect of feigning amnesia (e.g., Christianson and Bylin, 1999; van Oorsouw and Merckelbach, 2004). On the one hand, our findings might be related to the crime stimulus that was used (i.e., mock crime video). Indeed, a considerable body of research has demonstrated that visual stimuli are typically remembered better than verbal material (Nelson et al., 1976; Paivio, 1976, 1986; Weldon et al., 1989; Mintzer and Snodgrass, 1999). Hence, the use of a video could have led to a more solid memory trace for the mock crime information. Relatedly, on the other hand, perhaps simulators might have processed crime-related information more elaborately than controls – who did not receive any instructions after the mock crime viewing – in order to come up with a personal simulated version of the offense. In fact, although one could expect that feigning amnesia mainly leads to omitting information, previous studies suggested that when participants were asked to recall a mock crime in such a way they had great difficulties in remembering what happened, they were even likely to provide an alternative self-generated story (e.g., van Oorsouw and Merckelbach, 2004, 2006; van Oorsouw and Giesbrecht, 2008). Indeed, van Oorsouw and Merckelbach (2006) found that one-third of their entire sample used an alternative story in an attempt to feign amnesia. Yet, this result appears to be in line with the idea that by enhancing an active elaboration of information during memory encoding (McWilliams et al., 2014), being tested in itself could promote correct recollection (Chan, 2010; Fazio et al., 2010). However, even though feigners were more accurate than controls, no significant differences were found between groups on the number of correct responses provided during the cued recall test. This may suggest that individuals who did not receive any instruction during the first memory phase might find it easier to report correct information when prompted by open-ended cued recall questions rather than through a free recollection (Craik and McDowd, 1987; Padilla-Walker and Poole, 2002).

Finally, regarding our third and fourth hypothesis, no significant differences were found between simulators and confessors with respect to both distortion and commission errors during free and cued recall tests. This result might indicate that feigners recovered up to the level of confessors when it concerned distorted or self-generated information. Of interest, simulators increased distortions from T1 to T2 on the free recollection test. Conceivably, when participants instructed to feign amnesia come up with a self-generated version of the crime, which is strongly related to the original event, distortions are more likely to occur (Chrobak and Zaragoza, 2008; van Oorsouw and Giesbrecht, 2008; Otgaar and Baker, 2017).

A subsidiary aim of the present study was to explore whether or not inner speech (Alderson-Day and Fernyhough, 2015) might work as a buffer against the memory-undermining effect of feigning amnesia. One could argue that the more simulators tend to think of their crime, the less simulating amnesia affects the actual memory of the offense. However, our analyses suggest that inner speech might not be involved in preserving the genuine memory for the crime.

In sum, we suggest that feigning amnesia in the first place might be seen as a way to preserve and perhaps enhance memory for the target event over time, as compared to not being initially interviewed. However, possible memory decrements for feigners might depend on simulating amnesia in itself rather than a mere lack of rehearsal (e.g., Christianson and Bylin, 1999; van Oorsouw and Merckelbach, 2004). That is, drawing on the Memory and Deception (MAD) framework (Otgaar and Baker, 2017), feigning amnesia is inserted in a lying-continuum from false denial to fabrication of alternative scenarios. According to the MAD, the amount of cognitive resources required by individuals is directly proportional to the type of lie exerted, and the memory outcome for the actual target event is strictly affected by the different lie adopted. More precisely, whereas false denial implies less cognitive resources leading to omission, fabricating entire stories *ex novo* requires more cognitive resources leading to commission errors (Otgaar and Baker, 2017). Therefore, located somewhere in the middle of this framework, one could expect that feigning amnesia might cause a more distinct memory-undermining effect on the original experience by mostly withholding information than feigning amnesia by distorting or self-generating new information might do. In this latter case, perhaps due to a cognitive re-elaboration of the event (McWilliams et al., 2014), simulating amnesia might partially affect genuine memory for the crime even though participants might potentially report distortion and/or commission errors (e.g., Chrobak and Zaragoza, 2008; van Oorsouw and Giesbrecht, 2008; Otgaar and Baker, 2017).

Also, it could be argued that the instructions given to participants to feign amnesia for a mock crime might play a role in the actual memory for those events. As rightly noticed by Otgaar and Baker (2017), when those asked to feign amnesia account for the target experience, both attempts at simulating memory loss and fabricating may be occurring. Therefore, future studies might consider to bypass the “feigning” instruction by instead instructing participants to avoid thinking of the crime as a consequence of an emotional distress. Such deliberate avoidance, which would produce forgetting in itself as some research has pointed out (e.g., Anderson and Green, 2001; Anderson and Hanslmayr, 2014), might better reflect memory processes in real offenders.

Several caveats of the present study need to be mentioned. Although we tested a large number of participants, our sample mainly consisted of women. Even though we asked participants to identify themselves with the main character in the video, it might be hard for women to identify themselves with a male offender. To avoid this limitation in future research, it might be better to use a mock crime video recorded in *point of view* (pov) in which the perpetrator’s gender is indistinguishable. Moreover, we did not specifically assess participants’ ability to identify with the offender, which represents a limitation. Secondly, we did not assess the emotional impact of the mock crime video on participants. Specifically, we do not know whether or not the material used had contributed to maintain the participants’ memory performance over time *per se* regardless of the instruction given at T1. In future studies, therefore, it would be wise to assess the emotional impact of the crime material. Moreover, in future research, it might be interesting comparing two or more different types of crime materials to identify the most suitable stimulus to use in the mock crime paradigm (e.g., video vs. narrative story). It could be the case that the memory-undermining effect of simulating amnesia, that already seems to be less solid by adopting a mock crime video rather than a narrative, would perhaps be even weaker with a more ecologically valid set-up (e.g., mock crime through virtual reality). Thirdly, we did not ask participants what type of strategy they adopted to come up with a simulated version of the crime. Certainly, this information would be interesting since it is not fully clear to which degree this detrimental effect on the genuine memory for a crime is due to the feigning amnesia in itself or to the act of self-generating an alternative scenario for the same target event (Otgaar and Baker, 2017). Finally, another limitation has to do with the VISQ inner speech scales and the lack of correlation between these latter and the memory-undermining effect of feigning amnesia. Arguably, for this specific measure our sample size (i.e., 37 feigners) may not have been large enough to detect the predicted correlation. Future research in this direction should involve a larger sample size to better investigate a potential relation between the simulating amnesia effect and the inner speech traits. Moreover, even though this instrument has satisfactory psychometric reliability, the VISQ in its present form does not tap traits such as cognitive functions (McCarthy-Jones and Fernyhough, 2011). These functions – mnemonic and attentional uses of inner speech – are, however, assessed by other instruments which were not used in the present study (e.g., Self-Verbalization Questionnaire; Duncan and Cheyne, 1999). In future studies it would be wise to tap participants’ inner speech activities through self-report measures instead of mainly assessing their inner speech predisposition.

Although it is always difficult to generalize experimental findings to real life cases (Schacter, 1986), research using laboratory mock crime scenarios are fundamental to increase our knowledge about crime-related amnesia (e.g., McWilliams et al., 2014). As a matter of fact, knowing that genuine memory of a crime might be largely uncompromised despite having previously feigned amnesia appears to be informative to forensic practitioners who are asked to provide an opinion concerning crime-related amnesia cases. Relatedly, police investigators might find it interesting that suspects might actually preserve memory for the crime and contribute to disclose specific crime-related details. Oftentimes, indeed, crucial information of crimes remain undisclosed when a report of amnesia emerges (van Oorsouw and Merckelbach, 2004, 2006), regardless of the fact that some perpetrators admit their guilt (Porter et al., 2001). Thus, at least to some degree, our findings suggest that perpetrators are more likely to recall a larger amount of information when prompted by cues rather than being asked to freely recall the crime (see also Meissner et al., 2012; Mangiulli et al., unpublished, Study 2), particularly when they might be persuaded to collaborate with the justice department (e.g., plea bargaining situation).

In closing, by using a mock crime video instead of a mere narrative, our findings suggest that the memory-undermining effect of simulating amnesia occurs to a lesser extent than that observed in previous research (e.g., Christianson and Bylin, 1999; van Oorsouw and Merckelbach, 2004, 2006). The present study, indeed, indicates that simulating amnesia partially undermines actual memory for a crime and that, apart from a tendency to distort some details, offenders might still have relatively intact memory for the target experience.

## Author Contributions

IM conceived of the presented idea supported by HM, designed and directed the study in collaboration with KO, processed the experimental data and performed the analysis assisted by AC, and wrote the manuscript with input from all co-authors. MJ supervised the project.

## Conflict of Interest Statement

The authors declare that the research was conducted in the absence of any commercial or financial relationships that could be construed as a potential conflict of interest. The reviewer JS declared a past co-authorship with one of the authors HM to the handling Editor.

## Footnotes

1 The five subscales of the SIMS (Smith and Burger, 1997) were not included in our analyses since they were not relevant for our study.

2 None of our participants belonging to either one of the three groups (simulators vs. confessors vs. controls) were excluded from our study based on their SIMS results.

3 Additionally, we conducted similar ANOVAs on the free recall accuracy scores. Accuracy scores were calculated by dividing the correctness scores by the number of correct items plus the number of distortion and commission errors [Accuracy = correctness score/(correctness score + errors)]. Overall, findings showed that simulators were less accurate than confessors during T1 (*M_simulators_* = 59%, *SD* = 0.28 vs. *M_confessors_* = 81%, *SD* = 0.18), *t*(69) = -4.23, *p* < 0.001, *d* = 0.95. Moreover, at T2, confessors resulted to be more accurate than both simulators (*M_confessors_* = 86%, *SD* = 0.13 vs. *M_simulators_* = 72%, *SD* = 0.18) and controls (*M_controls_* = 67%, *SD* = 0.28), *p* = 0.02, 95% CI [0.02 0.26], *d* = 0.87, and *p* < 0.001, 95% CI [0.07 0.31], *d* = 0.87, respectively. No significant differences were found between simulators and controls, *p* = 0.85, 95% CI [-0.06 0.17].

4 We furthermore calculated the cued recall accuracy scores [Accuracy = correctness score/(correctness score + errors)]. Simulators appeared to be less accurate than confessors at T1 (*M_simulators_* = 63%, *SD* = 0.20 vs. *M_confessors_* = 75%, *SD* = 0.13), *t*(72) = -3.21, *p* = 0.002, *d* = -0.75. Yet, during T2, both simulators and confessors were more accurate than controls (*M_simulators_* = 75%, *SD* = 0.12, and *M_confessors_* = 75%, *SD* = 0.15, vs. *M_controls_* = 66%, *SD* = 0.13), *p* = 0.03, 95% CI [0.01 0.16], *d* = 0.65, and *p* = 0.02, 95% CI [0.01 0.17], *d* = 0.62, respectively. Finally, no significant differences were found between simulators and confessors, *p* = 1.00, 95% CI [-0.08 0.07].
